# Bone mineral density and content during weight cycling in female rats: effects of dietary amylase-resistant starch

**DOI:** 10.1186/1743-7075-5-34

**Published:** 2008-11-26

**Authors:** John D Bogden, Francis W Kemp, Abigail E Huang, Sue A Shapses, Hasina Ambia-Sobhan, Sugeet Jagpal, Ian L Brown, Anne M Birkett

**Affiliations:** 1 Trace Element and Mineral Research Laboratory, Department of Preventive Medicine and Community Health, UMDNJ-New Jersey Medical School, Newark, NJ, 07103-2714, USA; 2 Department of Nutritional Sciences, Rutgers University, New Brunswick, NJ 08901, USA; 3 Faculty of Health and Behavioural Science, University of Wollongong, Wollongong, NSW 2522, Australia; 4 GTC Nutrition, Golden, CO 80401, USA

## Abstract

**Background:**

Although there is considerable evidence for a loss of bone mass with weight loss, the few human studies on the relationship between weight cycling and bone mass or density have differing results. Further, very few studies assessed the role of dietary composition on bone mass during weight cycling. The primary objective of this study was to determine if a diet high in amylase-resistant starch (RS_2_), which has been shown to increase absorption and balance of dietary minerals, can prevent or reduce loss of bone mass during weight cycling.

**Methods:**

Female Sprague-Dawley (SD) rats (n = 84, age = 20 weeks) were randomly assigned to one of 6 treatment groups with 14 rats per group using a 2 × 3 experimental design with 2 diets and 3 weight cycling protocols. Rats were fed calcium-deficient diets without RS_2 _(controls) or diets high in RS_2 _(18% by weight) throughout the 21-week study. The weight cycling protocols were weight maintenance/gain with no weight cycling, 1 round of weight cycling, or 2 rounds of weight cycling. After the rats were euthanized bone mineral density (BMD) and bone mineral content (BMC) of femur were measured by dual energy X-ray absorptiometry, and concentrations of calcium, copper, iron, magnesium, manganese, and zinc in femur and lumbar vertebrae were determined by atomic absorption spectrophotometry.

**Results:**

Rats undergoing weight cycling had lower femur BMC (p < 0.05) and marginally lower BMD (p = 0.09) than rats not undergoing weight cycling. In comparison to controls, rats fed RS_2 _had higher femur BMD (p < 0.01) and BMC (p < 0.05), as well as higher values for BMD and BMC measured at the distal end (p < 0.001 and p < 0.01) and femoral neck (p < 0.01 and p < 0.05). Consistent with these findings, RS_2_-fed rats also had higher femur calcium (p < 0.05) and magnesium (p < 0.0001) concentrations. They also had higher lumbar vertebrae calcium (p < 0.05) and magnesium (p < 0.05) concentrations.

**Conclusion:**

Weight cycling reduces bone mass. A diet high in RS_2 _can minimize loss of bone mass during weight cycling and may increase bone mass in the absence of weight cycling.

## Background

Although obesity has a number of well-documented adverse effects on health, obese men and women have higher bone mineral density (BMD) and bone mineral content (BMC) than their age and gender-matched counterparts with lower body weights [1]. Weight cycling is the repeated loss and regain of body weight, and occurs frequently in obese and overweight men and women in their attempts to lose weight and maintain a lower body weight. Weight cycling may also occur in men and women of normal body weight or in those with eating disorders such as anorexia nervosa. The goal of intentional weight loss is to reduce fat mass, but there is typically an accompanying unwanted loss of lean body mass, including bone mass [1]. Although there is convincing evidence for a loss of bone mass with weight loss [1], there are only a few human studies on the relationship between weight cycling and bone mass or density. A small number of epidemiologic studies demonstrate that a history of weight cycling is associated with a reduction in bone mineral density or an increased risk of hip and other fractures in men and women [2-5]. In one of these studies [2], greater weight variability in a sample of about 20,000 women and 19,000 men was associated with relative risks of hip fracture of 2.07 for women and 2.70 for men. In another study of 169 premenopausal women [3], weight cycling was associated with significant (p < 0.01) decreases in BMD at the lumbar spine and distal radius. In the above studies, the significant relationships between weight cycling and hip fracture or BMD persisted even after correction for body weight. In more recent studies, Sogaard et al. [4] found that weight cycling in elderly Norwegian men was a risk factor for forearm fracture, and Bacon et al. [5] reported that chronic dieting in 30–45 year old obese women was associated with low bone mass. In contrast other investigators found that weight cycling did not reduce bone mass in competitive athletes [6] and overweight, sedentary, premenopausal women [7]. The varying results of the above and other human studies on the relationship between weight cycling and bone mass may be due to the many factors that cannot be adequately controlled in human studies, including subject diets, compliance with the study protocol, and patterns of weight loss. Furthermore, a history of weight cycling may define a subgroup of people at risk for bone loss due to other factors.

It is not known if dietary composition during weight cycling can influence bone mass, quality, and strength; specific diet components that may influence bone during weight cycling include vitamins D and K, calcium, magnesium, several essential trace minerals, and the resistant starches. Resistant starches (RS_1_, RS_2_, RS_3_, RS_4 _and RS_5_) are subgroups of fiber that occur naturally in foods or can be produced during food processing [8-11]. Total resistant starch is estimated to be about 10% (range = 2–20%) of ingested starches in Western diets [8]. The resistant starches are not digested by mammalian enzymes in the mouth, stomach, or small intestine, but may be fermented in the colon [8-11]. Categorization of the five types of resistant starch is based on the factors that explain their resistance to degradation in the upper gastrointestinal tract. These factors include physical entrapment of the starch by the native cellular structure (RS_1_), the physical structure of the starch granules (RS_2_), or their chemical composition (RS_3_, RS_4_, and RS_5_) (8–10). RS_2 _is the subtype most widely used commercially. We selected it for this investigation because a number of prior studies from other laboratories provide considerable evidence demonstrating that dietary RS_2 _improves absorption and balance of calcium, magnesium, and several trace minerals in experimental animals, particularly the rat [12-19]. This study is based on the hypothesis that RS_2_, by reducing gastrointestinal loss of calcium, magnesium, and other bone minerals, may help to maintain bone mass subsequent to weight cycling.

Because of the difficulty of controlling the various factors that could influence the effects of weight cycling on bone mass in humans, we conducted a study in rats so that diet composition, weight cycling patterns, age, and other relevant variables could be tightly controlled. The primary objective of this investigation was to determine if a diet high in natural RS_2 _from high-amylose cornstarch (also termed "maize starch") can reduce or prevent loss of femur BMD and BMC subsequent to weight cycling in female rats. The female Sprague-Dawley (SD) rat was chosen as the experimental model based on prior studies in our laboratories in this species; these studies suggest that the female SD rat is an appropriate model for the study of relationships between weight loss and bone composition [20-24]. A second goal was to analyze bone for other minerals and trace elements that are known or suspected to be required for optimal bone strength, specifically calcium, copper, iron, magnesium, manganese, and zinc [25]. This was done to obtain insight into specific bone metals that may be involved in the effects of weight cycling and/or dietary resistant starch on bone. A third goal was to analyze selected non-calcified organs (liver and kidney) with high metabolic activity for the same metals in order to compare effects of weight cycling and dietary resistant starch on bone versus soft tissue minerals.

## Methods

### Diet preparation

Diets were formulated and prepared by Research Diets, Inc., New Brunswick, NJ. The composition of these diets is described in Table 1. Carbohydrate in each diet is provided by waxy cornstarch, pregelatinized waxy cornstarch, and sucrose. A mixture of waxy cornstarch, pregelatinized waxy cornstarch, and sucrose was used for the control (normal) diets to provide a resistant-starch-free formulation capable of being formed into pellets. For the diets containing resistant starch, Hi-maize 260^® ^starch (National Starch and Chemical Company, Bridgewater, NJ) was substituted for a portion of the waxy cornstarch (Amioca, 0% amylose). Hi-maize 260 starch, a hydrothermally-treated RS_2 _starch composed of 60% total dietary fiber as RS_2_-type granules, is derived from cornstarch high in amylose but is resistant to enzymatic degradation by amylase. A target of 18–20% by weight of Hi-maize 260 starch was chosen because diets with a similar concentration of this component have been shown to improve retention of calcium, magnesium, and some trace minerals in the rat [17].

**Table 1:** Composition of calcium-deficient normal and resistant starch pelleted diets fed during weight maintenance/gain and during 40% energy restriction

	**Ad Lib**				**40% kcal Restricted**			
		
	**ND**		**RS**		**NDWL**		**RSWL**	
		
**Macronutrients**	**% gm**	**% kcal%**	**gm**	**% kcal**	**% gm**	**% kcal**	**% gm**	**% kcal**
		
Protein	19.5	21	18.4	21	19.2	21	18.2	21
Carbohydrate	67.3	64	69.1	64	66.3	64	68.1	64
Fat	6.2	15	5.9	15	6.2	15	5.8	15
Total	93.0	100	93.4	100	91.7	100	92.1	100
		
Energy/gram	**3.74 kcal/g**		**3.54 kcal/g**		**3.69 kcal/g**		**3.49 kcal/g**	
		
**Ingredients**	**gm**	**kcal**	**gm**	**kcal**	**gm**	**kcal**	**gm**	**kcal**

Casein, 80 Mesh	200	800	200	800	200	800	200	800
DL-Methionine	3	12	3	12	3	12	3	12
Waxy Cornstarch,	500.5	1752	361.5	1265	492.5	1724	354	1239
Pregelatinized Waxy Cornstarch	100	350	100	350	100	350	100	350
Sucrose	91	364	91	364	91	364	91	364
High Amylose Cornstarch	0	0	200	486	0	0	200	486
Cellulose, BW200	50	0	50	0	50	0	50	0
Corn Oil	65	585	65	585	65	585	65	585
AIN-76 Salt Mix, S10001A w/o Ca and P	17.5	0	17.5	0	29.2	0	29.2	0
Dibasic Calcium Phosphate, 29.5% Ca, 22.8% P	3.4	0	3.4	0	5.65	0	5.65	0
AIN-76A Vitamin Mix, V10001	10	40	10	40	16.7	67	16.7	67
Choline Bitartrate	2	0	2	0	3.3	0	3.3	0
FD&C Yellow Dye #5	0.05	0	0	0	0	0	0.025	0
FD&C Red Dye #40	0	0	0.05	0	0	0	0	0
FD&C Blue Dye #1	0	0	0	0	0.05	0	0.025	0

**Totals**	**1042.45**	**3903**	**1103.45**	**3902**	**1056.4**	**3902**	**1117.9**	**3903**

Waxy Cornstarch and Pregelatinized Waxy Cornstarch–3.5 kcal/g, High Amylose Cornstarch –2.43 kcal/g

ND = normal (control) diet fed *ad lib *during periods of weight maintenance or weight gain

RS = resistant starch diet fed *ad lib *during periods of weight maintenance or weight gain

NDWL = normal (control) diet fed during energy restriction/weight loss

RSWL = resistant starch diet fed during energy restriction/weight loss

The study was designed to ensure comparable dietary intake in all treatment groups during weight gain or weight loss of calcium, phosphorus, magnesium, and each of the essential vitamins and trace minerals. To achieve this goal, 4 diet formulations were required, as described in Table 1. The diets were formed into cylindrical pellets (22 mm × 12 mm in diameter) and colored with FD&C dyes to provide a distinct color for each diet formulation and facilitate conduct of the study by allowing easy identification of each of the four diets. Diet NDWL (normal diet for weight loss) was formulated to have concentrations of AIN-76 salt mix, calcium phosphate, AIN-76A vitamin mix, and choline bitartrate that were 40% higher than Diet ND (normal *ad lib *diet). Similarly, Diet RSWL (resistant starch (RS_2_) weight loss diet) was formulated to have concentrations of AIN-76 salt mix, calcium phosphate, AIN-76A vitamin mix, and choline bitartrate that were 40% higher than Diet RS (resistant starch (RS_2_) *ad lib *diet). Diets ND and RS were fed except when rats were undergoing energy restriction to achieve weight loss; during energy restriction diets NDWL or RSWL were fed.

The target calcium concentration for Diets ND and RS was 1.0 mg of calcium per gram of diet (0.1% calcium by weight = 25 μmol/g), a concentration that is 20% of the calcium concentration of the standard AIN-76 diet, and can be considered to be moderately deficient for the rat. This approach was used because many younger adults and more than 50% of older adults living in the United States, including those who use calcium-containing antacids or supplements, consume diets that are considered to be inadequate in calcium [26], and thus may be at higher risk of loss of bone mass during weight loss [27].

### Animal care and treatment

Female rats were chosen for this study because low BMD and BMC, as well as a higher risk of osteoporosis and osteoporosis-related fractures, are more common in women than men, and because of our prior experience studying effects of weight loss on bone in women and female rats [20-24].

The number of rats chosen per treatment group (n = 14) exceeds the number per group (as few as 6) in prior studies conducted in our laboratories [20-23] that demonstrate significant loss of bone mass after weight loss. In addition we conducted a power analysis based on the effect of a projected 18% loss of body weight on bone density in the rat. Using conservative assumptions and data from our prior studies, the power analysis showed that with 12 rats per treatment group and alpha = 0.05, there is 90% power to detect a difference in BMD between treatment groups of 4.7% and 80% power to detect a difference of 4.0%. Thus, it was anticipated that 14 rats per group would provide adequate statistical power to detect treatment effects of moderate size on the major outcome variables of BMD and BMC even with attrition of as many as 2 rats per group.

**Figure 1:**
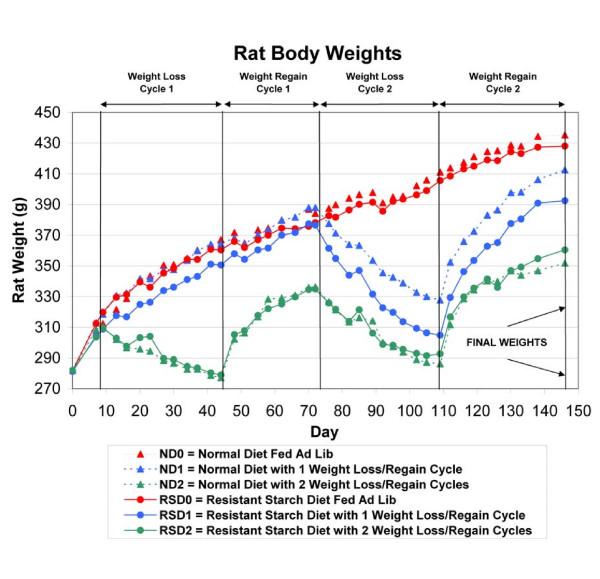
**Rat body weights**. Mean body weights of rats in the six treatment groups during the 21-week duration of the study. N = 84 rats. Each point in the figure is the mean for the 14 rats in each of the 6 treatment groups.

Sexually mature female Sprague-Dawley (SD) rats (Charles River, Raleigh, NC, n = 84, age = 20 weeks) were randomly assigned to one of six treatment groups with 14 rats per group using a 2 × 3 experimental design with 2 diets and 3 weight cycling protocols. Because body weight is strongly associated with bone mass, rats were stratified by body weight prior to random assignment to one of the 6 treatment groups to ensure nearly identical initial mean body weights in each of the 6 groups. As anticipated, this approach to random assignment resulted in very similar mean body weights at randomization (281.2–282.5 g) in each of the six treatment groups (Figure 1). Rats were fed pelleted diets without RS_2 _(ND = normal diets, controls) or diets high in RS_2 _(17.9–18.1%) throughout the study. The weight cycling protocols were: weight maintenance with no weight cycling (Groups ND0 and RSD0), 1 round of weight cycling (Groups ND1 and RSD1), or 2 rounds of weight cycling (Groups ND2 and RSD2).

Diets ND or RS were fed upon arrival of the rats in our laboratories for 8 days prior to the initiation of energy restriction/weight loss. The plan and chronology of weight loss and weight regain is illustrated in Figure 1. The timing of the single round of weight cycling for those rats undergoing this treatment coincided with the final (second) round of weight cycling in rats undergoing 2 rounds of weight cycling. Weight loss was achieved by restriction of energy intake to 60% of that of rats in groups fed *ad lib*, as in our prior studies [20,21,23]. All rats were allowed to consume feed *ad lib *when not undergoing weight loss.

Feed consumption was monitored by weighing all feed provided and subtracting feed not eaten. Feed consumed by rats eating *ad lib *during a week was used to calculate the reduced amounts of feed provided to rats consuming energy restricted diets during the following week. When rats were consuming diets *ad lib*, they were fed once per week. When rats were consuming energy restricted diets, they were fed daily including weekends, and invariably ate all feed provided.

Rats were housed throughout the study in individual cages in a separate, locked room in the New Jersey Medical School Research Animal Facility, which is AAALAC accredited. Cages and cage racks were labeled with tape of six different colors to facilitate identification of rats in each of the six treatment groups. No other animals were housed in the room during the study. Blood withdrawal was done in a nearby procedure room within the Research Animal Facility

After completion of the second round of weight regain after 21 weeks, blood was obtained by cardiac puncture under anesthesia with 0.6 ml/100 g of a mixture of ketamine (20 mg/ml) and xylazine (2.5 mg/ml) administered intraperitoneally. Rats were then euthanized by decapitation after administration of additional anesthetic if necessary. Both femurs and the spinal column bones of the lumbar region were harvested. Other organs harvested from each rat were liver and kidney. Procedures used to harvest the organs have been used on many occasions in our laboratory and have been designed to minimize the potential of organ metal contamination during harvesting. For the right femur to be used for measurement of BMD and BMC, femurs were preserved by wetting them with 50% physiological saline (0.42%) solution and wrapping them in cotton gauze saturated with 50% saline prior to freezing. All blood and organ samples were stored at -70°C until analysis.

### Laboratory analyses

After placing the excised femur on a Delrin block, BMC and BMD of the right femurs were determined by dual energy x-ray absorptiometry (DEXA) using a GE-Lunar PIXImus densitometer with software version 1.4×. The PIXImus densitometer uses a 14 degree stationary anode X-ray tube generator with a 0.25 mm × 0.25 mm focal spot that generates a cone beam X-ray (55/80 kVp at 400 μA). The right femurs were analyzed for the whole bone "total" BMD and BMC, and at 2 other standard sites prone to fracture, specifically the femoral neck and the distal femur (20% from the distal end of the bone) [28].

After non-destructive analysis by DEXA, the right femurs were subsequently used for laboratory analyses of their mineral composition; this enabled measurement of mineral concentrations using the same bone that was used to determine BMD and BMC. The right femur and lumbar vertebrae bones were analyzed for minerals and trace elements that are considered to be essential or beneficial for optimal bone strength, specifically calcium, copper, magnesium, manganese, and zinc [25]. Iron was also determined in these samples. For these analyses the entire right femur and a set of 3–4 lower lumbar vertebrae were assayed; the bone samples were cleaned of all non-calcified tissue before analysis. The non-calcified organs collected (kidney and liver) were also analyzed for the same metals. These analyses were conducted using flame or electrothermal atomic absorption spectrophotometry. Standards, blanks, and National Institute of Standards and Technology (NIST, Gaithersburg, MD) Standard Reference Materials (SRM # 1486–Bone Meal or SRM # 1577b–Bovine Liver) with certified values for their metal concentrations were analyzed with each set of daily analyses as a component of our quality control program [20,21].

### Data analysis

Data evaluation methods included descriptive and inferential statistical analyses. Two-way ANOVA was conducted to determine the separate effects of dietary resistant starch and weight cycling, as well as their interactions, on the various study outcome variables. Pairwise differences were evaluated subsequent to 2-way ANOVA by comparison of least-square means at p < 0.05. Associations between selected variables were determined by calculation of Pearson correlation coefficients. A limited number of multiple regression analyses were conducted with BMD or BMC as the dependent variable and final body weight and treatment group as the independent variables.

### Ethical treatment of animals

All applicable institutional and governmental regulations concerning the ethical use of animals were followed during this research. The study and its specific procedures were reviewed and approved by the UMDNJ-New Jersey Medical School Institutional Animal Care and Use Committee.

## Results

### Food consumption

Rats fed the normal diets and their counterparts fed the resistant starch diets had similar total food intakes (mean ± SE) during the 21-week study, specifically 2566 ± 97 g and 2585 ± 88 g for those rats not undergoing weight cycling, 2333 ± 59 g and 2424 ± 53 g for those rats subject to one round of weight cycling, and 2142 ± 31 g and 2118 ± 55 g for those rats undergoing two rounds of weight cycling.

### Diet composition

Table 2 contains the results of our analyses of diet concentrations of iron and the 5 metals for which there is evidence that they are essential (Ca) or likely to be important (Cu, Mg, Mn, Zn) in the maintenance of bone mineral density and bone mineral content. Our target for diets moderately deficient in calcium for the rat was a concentration of 1.0 mg of calcium per gram of diet (25.0 μmol/g). Measured mean calcium concentrations for Diets ND and RS fed during periods of weight maintenance or weight gain were 1.09 mg/g (27.2 μmol/g) and 1.07 mg/g (26.7 μmol/g), and thus are within 10% of the target concentration. Calcium present in other food components besides the dibasic calcium phosphate used to add calcium to the diets likely contributed to the total calcium concentration of the diets. Diets NDWL and RSWL that were fed during periods of weight loss at 60% of ad lib were intended to have calcium concentrations that were 40% (1.40-fold) greater than Diets ND and RS; measured values were 51% and 42% greater, respectively. The latter results are based on 8 replicate analyses, but even well mixed solid diets may have some degree of heterogeneity. Measured concentrations of Cu, Fe, Mg, Mn, and Zn in Diets ND and RS were near the target levels for the AIN-76 diet for rodents that are provided in Table 2.

**Table 2:** Essential mineral concentrations of custom calcium-deficient diets

**Diet**	**Calcium μmol/g**	**Copper μmol/g**	**Iron μmol/g**	**Magnesium μmol/g**	**Manganese μmol/g**	**Zinc μmol/g**
ND	27.20 ± 0.44	0.094 ± 0.004	0.775 ± 0.018	21.39 ± 0.13	0.978 ± 0.020	0.577 ± 0.006
RS	26.70 ± 0.53	0.092 ± 0.001	0.736 ± 0.022	20.56 ± 0.21	0.923 ± 0.011	0.539 ± 0.007
						
NDWL	41.17 ± 0.88	0.136 ± 0.004	1.074 ± 0.007	34.14 ± 0.40	1.429 ± 0.010	0.809 ± 0.008
RSWL	37.92 ± 0.76	0.134 ± 0.002	1.043 ± 0.021	32.49 ± 0.18	1.372 ± 0.027	0.780 ± 0.008

**AIN 76**	**129.7**	**0.094**	**0.63**	**20.6**	**1.07**	**0.55**

Ratio:						
NDWL/ND	1.51	1.44	1.39	1.60	1.46	1.40
RSWL/RS	1.42	1.45	1.42	1.58	1.49	1.45

ND = normal diet fed *ad lib *during periods of weight maintenance or weight gain

RS = resistant starch diet fed *ad lib *during periods of weight maintenance or weight gain

NDWL = normal diet fed during periods of weight loss

RSWL = resistant starch diet fed during periods of weight loss

AIN 76 = concentrations in the standard American Institute of Nutrition 1976 Rodent Diet

Concentrations are mean ± SE N = 8 for all values

### Body weights

Figure 1 depicts mean body weights for the 14 rats in each of the six treatment groups. These data demonstrate that we were able to successfully produce weight loss and regain in the groups of rats undergoing one round of weight cycling (ND1 and RSD1) or two rounds of weight cycling (ND2 and RSD2). The results further demonstrate that body weights in the normal diet and corresponding resistant starch diet groups (ND0 versus RSD0 and ND2 versus RSD2) tracked closely with one another throughout the study. Final body weights differed slightly between the ND0 and RSD0 groups (7.3 g = 1.7% difference in mean final body weights), and the ND2 and RSD2 groups (8.6 g = 2.5% difference in mean final body weights). Final body weights differed more substantially between the ND1 and RSD1 groups (20.1 g = 5.1% difference in mean final body weights). Despite random assignment of rats to the six treatment groups and virtually identical mean group body weights subsequent to random assignment to groups (see Figure 1, Day 0), the body weights of the latter two groups did not track as closely as the other groups described above (see Figure 1).

### Bone mineral density & content

**Figure 2:**
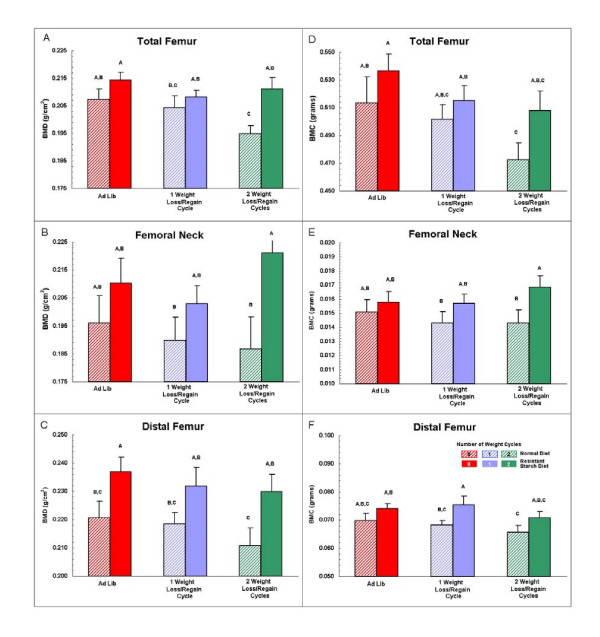
**Bone mineral density and bone mineral content**. Bone mineral density (BMD) and bone mineral content (BMC) of the total femur, femoral neck, and distal femur for right femurs of rats in the six treatment groups after 21 weeks. N = 84 rats. Each bar in the figure is the mean with standard error for 14 femurs. Mean BMD and BMC are consistently higher for rats fed the diets containing resistant starch (solid bars) than for their counterparts (adjacent striped bars) fed the normal diets. Values not marked with the same upper-case letters (A, B, or C) differ significantly (p < 0.05) by comparison of least-square means. Inferential statistical analyses of these data by 2-way ANOVA are provided in Table 3.

Figure 2 contains the results for BMD and BMC for right femurs of the rats. Results are provided for the BMD and BMC of the entire femur, as well as for the distal femur and femoral neck, for a total of six variables. Pairwise comparisons in Figure 2 reveal that the values for BMD and BMC were always higher (total of 18 pairwise comparisons) in rats fed the diets containing resistant starch than in the corresponding rats fed the normal diets without added resistant starch (ND0 versus RSD0, ND1 versus RSD1, ND2 versus RSD2). Mean values for BMD-total in the RSD0, RSD1, and RSD2 groups were 3.4%, 2.0%, and 8.2% higher, respectively, than those of corresponding rats in the ND0, ND1, and ND2 groups. Values were similarly higher for BMD-femoral neck (7.1%, 6.8%, and 18.2%) and BMD-distal femur (7.2%, 6.4%, and 9.0%) in the RSD groups than the corresponding ND groups. The same pattern was observed for percentage differences in BMC between the RSD versus ND groups. The above results show that BMD and BMC were consistently higher in rats fed the resistant starch diets than in their counterparts fed the control diets; this result cannot be explained by differences in body weights between rats fed the resistant starch diets and their counterparts fed the normal diets because their body weights were very similar throughout the study (Figure 1).

Pearson correlation coefficients for the associations between BMD-total, BMD-femoral neck, and BMD-distal femur and the corresponding values for BMC were 0.818, 0.974, and 0.875 (n = 83–84, p < 0.0001).

We evaluated the results for BMD and BMC using 2-way ANOVA (Table 3) to determine the independent effects of diet (RS_2 _versus ND diet) and weight cycling protocol (0, 1, or 2 rounds of weight cycling), as well as the effect of diet/weight cycling interactions, on these variables. Compared to rats fed the normal diet, rats fed the RS_2 _diet had significantly higher BMD-total, BMD-distal femur, and BMD-femoral neck; the corresponding values for BMC were also significantly higher in RS_2 _-fed rats. Rats undergoing weight cycling had significantly lower BMC-total than rats not undergoing weight cycling. Weight cycling did not significantly affect the other 5 measures of BMD and BMC, although the effect of weight cycling on BMD-total approached statistical significance (p = 0.09). Weight cycling and diet did not interact to significantly influence any measure of BMD or BMC.

**Table 3:** Bone mineral density and content of total femur, femoral neck and distal femur: P values for 2-way ANOVA

	**Diet Effect**	**Weight Cycling Effect**	**Diet/Weight Cycling Interaction**
BMD Total Femur	p < 0.01	NS^a^	NS
BMD Femoral Neck	p < 0.01	NS	NS
BMD Distal Femur	p < 0.001	NS	NS

BMC Total Femur	p < 0.05	p < 0.05	NS
BMC Femoral Neck	p < 0.05	NS	NS
BMC Distal Femur	p < 0.01	NS	NS

NS = ≥0.05

Results are for the right femur

^a^p = 0.09

Diet Effect = normal diets versus resistant starch diets

Weight Cycling Effect = 0, 1, or 2 rounds of weight cycling

Figure 1 shows that rats in the four treatment groups undergoing weight cycling (ND1, ND2, RSD1, RSD2) had mean body weights that at their minimum values were about 80–120 g lower than those of rats not undergoing weight cycling (groups ND0 and RSD0). Rats undergoing weight cycling also had substantially lower final body weights. As expected, final body weights were significantly associated with BMD-total (r = 0.32, p = 0.003) and with BMC-total (r = 0.46, p < 0.0001). Therefore, to further define relationships among BMD, final body weight, and treatment group, we conducted a multiple regression analysis. In this model BMD-total was the dependent variable and final body weight and treatment group were the independent variables. Final body weight and treatment group were significant (p < 0.01) predictors of BMD-total, accounting for 18.7% of the variability in BMD-total (final body weight = 10.3% of variability, treatment group = 8.3% of variability).

### Mineral concentrations

None of the kidney metal concentrations measured differed significantly among treatment groups. For liver there were some differences in concentrations among treatment groups, but no consistent relationships to weight cycling or diet composition were observed.

Table 4 contains mineral concentrations for femur and lumbar vertebrae bone samples. We evaluated these results for femur mineral concentrations using 2-way ANOVA (Table 5) to determine the independent effects of diet (RS_2 _versus ND diet) and weight cycling protocol (0, 1, or 2 rounds of weight cycling), as well as diet/weight cycling interactions, on these variables. Compared to controls fed the normal diet, rats fed the RS_2 _diet had significantly higher femur calcium and magnesium concentrations, but did not have higher femur concentrations of copper, iron, manganese, and zinc. Weight cycling had no significant effect on femur metal concentrations of calcium, copper, manganese, and magnesium. However, rats undergoing weight cycling had significantly lower femur zinc, and there were significant interactions between weight cycling and diet that influenced femur iron and zinc concentrations.

**Table 4:** Calcified tissue mineral concentrations

**RIGHT FEMUR**						
**Group**	**Calcium mmol/g**	**Copper nmol/g**	**Iron μmol/g**	**Magnesium mmol/g**	**Manganese nmol/g**	**Zinc μmol/g**

ND0	3.98 ± 0.16	9.33 ± 0.55	0.98 ± 0.08	0.118 ± 0.002	11.23 ± 0.57	3.25 ± 0.08
ND1	4.17 ± 0.10	9.44 ± 0.39	1.23 ± 0.10	0.119 ± 0.002	11.30 ± 0.65	2.94 ± 0.07
ND2	4.06 ± 0.07	8.42 ± 0.37	1.15 ± 0.09	0.120 ± 0.002	11.18 ± 0.55	2.81 ± 0.05
						
RSD0	4.22 ± 0.06	8.83 ± 0.37	1.52 ± 0.15	0.126 ± 0.002	11.61 ± 0.67	2.98 ± 0.07
RSD1	4.26 ± 0.07	8.64 ± 0.36	1.11 ± 0.09	0.127 ± 0.002	10.92 ± 0.79	3.05 ± 0.05
RSD2	4.30 ± 0.09	8.62 ± 0.38	1.10 ± 0.07	0.127 ± 0.002	10.45 ± 0.71	2.96 ± 0.05

**LUMBAR VERTEBRAE**						

**Group**	**Calcium mmol/g**	**Copper nmol/g**	**Iron μmol/g**	**Magnesium mmol/g**	**Manganese nmol/g**	**Zinc μmol/g**

ND0	4.25 ± 0.12	10.22 ± 0.53	1.19 ± 0.10	0.126 ± 0.002	8.14 ± 0.28	3.25 ± 0.07
ND1	4.26 ± 0.09	10.36 ± 0.69	1.43 ± 0.10	0.123 ± 0.003	8.68 ± 0.45	3.17 ± 0.09
ND2	4.31 ± 0.07	10.84 ± 0.88	1.25 ± 0.06	0.128 ± 0.003	8.11 ± 0.27	3.20 ± 0.06
						
RSD0	4.48 ± 0.06	10.26 ± 0.49	1.63 ± 0.21	0.132 ± 0.003	8.53 ± 0.33	3.30 ± 0.06
RSD1	4.60 ± 0.23	11.50 ± 0.83	1.22 ± 0.04	0.130 ± 0.003	8.34 ± 0.35	3.27 ± 0.06
RSD2	4.44 ± 0.08	9.84 ± 0.55	1.16 ± 0.07	0.131 ± 0.002	7.70 ± 0.31	3.21 ± 0.09

Values are Mean ± SE based on wet organ weight, n = 14 for all tabular values except that n = 8 for femur copper.

Inferential statistical analyses of these data by 2-way ANOVA are provided in Table 5 for the right femur and Table 6 for the lumbar vertebrae.

ND0 = normal diet with no weight cycling

ND1 = normal diet with 1 round of weight cycling

ND2 = normal diet with 2 rounds of weight cycling

RSD0 = resistant starch diet with no weight cycling

RSD1 = resistant starch diet with 1 round of weight cycling

RSD2 = resistant starch diet with 2 rounds of weight cycling

**Table 5:** Femur mineral concentrations: P values for 2-way ANOVA

**Metal**	**Diet Effect**	**Weight Cycling Effect**	**Diet/Weight Cycling Interaction**
Ca	p < 0.05	NS	NS
Cu	NS	NS	NS
Fe	NS	NS	p < 0.01
Mg	p < 0.0001	NS	NS
Mn	NS	NS	NS
Zn	NS	p < 0.01	p < 0.01

NS = p ≥ 0.05

Results are for the right femur

Diet Effect = normal diets versus resistant starch diets

Weight Cycling Effect = 0, 1, or 2 rounds of weight cycling

Compared to controls fed the normal diet, data evaluation using 2-way ANOVA demonstrated that rats fed the RS_2 _diet had significantly higher lumbar vertebrae calcium and magnesium concentrations, but did not have higher concentrations of copper, iron, manganese, and zinc (Table 6); these results are consistent with the results for the femur metal concentrations. Weight cycling did not significantly affect any of the 6 lumbar vertebrae metal concentrations measured; however, as observed for the femur, there was a significant interaction between weight cycling and diet that influenced lumbar vertebrae iron concentrations.

**Table 6:** Lumbar vertebrae mineral concentrations: P values for 2-way ANOVA

**Metal**	**Diet Effect**	**Weight Cycling Effect**	**Diet/Weight Cycling Interaction**
Ca	p < 0.05	NS	NS
Cu	NS	NS	NS
Fe	NS	NS	p < 0.05
Mg	p < 0.05	NS	NS
Mn	NS	NS	NS
Zn	NS	NS	NS

NS = p ≥ 0.05

Diet Effect = normal diets versus resistant starch diets

Weight Cycling Effect = 0, 1, or 2 rounds of weight cycling

Femur calcium, magnesium, and zinc concentrations were significantly associated with BMD-total (r = 0.24, 0.29, and 0.23, p < 0.05), but femur copper, iron, and manganese were not (r = 0.04, 0.00, and 0.01, p > 0.05). Similarly, femur calcium, magnesium, and zinc concentrations were significantly associated with BMC-total (r = 0.28, 0.30, and 0.25, p < 0.05), but femur copper, iron, and manganese were not (r = 0.02, 0.00, and 0.01, p > 0.05).

Using the femur calcium concentrations and femur weights, we calculated the total calcium content of the right femurs. These values were significantly associated with BMC-total of the right femur (r = 0.943, p < 0.0001), demonstrating very good agreement between the DEXA measurements of BMC-total done in the laboratory of one of the co-authors (SAS) and femur calcium concentrations measured by flame atomic absorption spectrophotometry in the laboratory of another co-author (JDB).

## Discussion

The results provide the first evidence that a dietary component, in this case RS_2_, can prevent or reduce loss of BMD and BMC due to weight cycling. Rats fed RS_2 _also had higher bone mass in the absence of weight cycling. Although the differences we found among treatment groups in BMD and BMC are small-to-moderate in magnitude (2.8–18.2%), they are within the range of the decreases in BMD and BMC of about 10% that are associated in women with a substantially increased risk of fractures of the hip, wrist, or spinal column [29,30].

Although BMD and BMC for the total femur, distal femur, and femoral neck were consistently lower in rats undergoing weight cycling in comparison to those animals not losing weight, the differences were significant (p < 0.05) only for BMC total. Because we measured BMD and BMC only after regain of body weight, larger reductions in BMD and BMC with weight loss may have been partially restored by the rapid regain of body weight prior to harvesting the femurs for determination of BMD and BMC.

In the current study rats fed the RS_2 _diet had significantly higher femur calcium and magnesium concentrations. We also found significant pairwise associations between femur BMD or BMC and femur calcium and magnesium concentrations. These data suggest that dietary RS_2 _may prevent loss of bone mass during weight cycling by enhancing absorption and retention of calcium and/or magnesium (as suggested by the Table 5 data for femur calcium and magnesium), but not the retention of copper, iron, manganese, and zinc. This result is consistent with the results of prior studies [16,20] showing that RS_2 _increases calcium and magnesium absorption; a potential mechanism for this effect is hypothesized to be the cecal wall hypertrophy and cecal acidification that RS_2_-induced fermentation produces in the large intestine [19].

Lobo et al. [31] have studied the effect of a diet containing 5% fructooligosaccharides (FOS) on intestinal absorption of calcium and magnesium, bone mineral density, and bone strength parameters in growing 4-week old male rats. Compared to controls, rats fed *ad lib *with FOS had a higher apparent absorption of calcium and magnesium, higher femur and tibia calcium concentrations, and increased femur peak load and yield load. Thus, our study and that of Lobo et al. suggest that specific dietary carbohydrates may have important roles in building and maintaining bone mass and strength during weight cycling or growth, and that the mechanism for this effect may be enhanced calcium and magnesium absorption and retention.

A recent study [32] used NHANES III data to estimate the mean intake of total resistant starch in the diets of USA residents age one year or older. The mean intake was 4.9 g/day, with a range of 3–8 g/day. The major sources of resistant starch were breads (21%), cooked cereals and pasta (19%), vegetables other than legumes (19%), bananas/plantains (14%), and legumes (9%). Legumes contained the most resistant starch per serving, as much as 8 grams. Comparison of the above intakes for an adult to the RS_2 _dose administered to the rats in the current study suggests that higher intakes than those found for the above USA residents studied may be needed to effect short-term improvements in BMD and BMC. However, it is possible that long-term consumption of intakes at the high end of the range of 3–8 g of total resistant starch daily may improve BMD and BMC.

In the current study 20-week old female rats were fed diets that were deficient in calcium but that contained levels of copper, iron, magnesium, manganese, and zinc that were near AIN-76 target values. Thus, the diets contained normal/adequate concentrations of the latter five essential metals that are considered to be important for bone health. The effects of RS_2 _on BMD and BMC might differ from the results found in the current study if the diets were adequate in calcium or deficient in copper, iron, magnesium, manganese, and/or zinc. The results might also differ if male rats or female rats of a different age were studied, because age and gender have substantial effects on BMD and BMC and on bone metabolism.

## Conclusion

Weight cycling reduces bone mass. However, a diet high in RS_2 _can prevent or reduce loss of bone mass during weight cycling and may increase bone mass in the absence of weight cycling. The results provide the first evidence that a dietary component can help preserve BMD and BMC during weight cycling. If the above effects of dietary RS_2 _are found to apply to humans, then increased dietary RS_2 _may help to build and preserve BMD and BMC in men and women, especially during weight cycling.

## Competing interests

Drs. Birkett and Brown were employees of the National Starch and Chemical Company for a portion of the period during which this research was being done. Neither is currently employed by the company.

## Authors' contributions

JDB designed and secured funding for this study, performed some of the laboratory work, and prepared the manuscript. FWK did some of the laboratory work and performed most of the data quality control efforts and statistical analyses. AEH, HA-S, and SJ did a considerable portion of the laboratory work. SAS, ILB, and AMB contributed to study design modifications, data evaluation, and manuscript revision.
